# Honors Properly Bestowed

**Published:** 1855-01

**Authors:** 


*Honors Properly Bestowed.*

It is well known that during the latter part of the past summer, the city of Savannah, Ga., was terribly scourged by yellow fever. When the epidemic was at its height, several of the resident physicians fell victims to it, and to supply their places others left their homes and volunteered their services. Among these were Drs. Hamilton and Redwood, of Mobile, and Dr. Cross. of New Orleans. Recently a meeting was held over which the Mayor presided, and each of the gentlemen named was presented with a service of plate, as a token of gratitude on the part of the citizens. At the same meeting resolutions were adopted commending the conduct of the resident physicians generally, and proposing to erect a monument to the memory of those who had fallen during the epidemic.

*Acne.*—For the cure of these troublesome and unseemly pimples which so often appear on the face, Cazenave has recently recommended the application of a solution of some of the Ammoniacal salts. The hydrochlorate or acetate may be used. He supposes the Ammonia to be capable of so acting on the fatty matter in the follicles as to convert it into a soap easy of solution and discharge

*The New Volume.*

The present number commences the 4th volume of the *New Series* of the North-Western Medical & Surgical Journal. We have not now, either time or disposition to review the past or speculate on the future. We wish merely to say that we commence the new year under arrangements which we believe will insure promptness and regularity in the issue of the Journal, and correctness in all its business relations. All the books and accounts of the Journal past and present have been transferred to N. S. Davis, one of the editors; and to him should all remittances, letters and papers be addressed. Whatever untiring industry and effort, on the part of the editors, can do to make the Journal one of the best in the United States, *shall* be done. But editors can no more make a good journal and keep it good without the prompt support of their patrons, than could the Israelites in Egypt, make bricks "without straw." Hence we hope our patrons will be prompt in furnishing both money and communications. The series of articles in relation to the causes and treatment of fevers, resumed in this number, will not occupy a place in every number, but will be completed, if possible in the present volume. The next number will contain an address on the 'Influence of Alcoholic drinks on man,' delivered by request, to the class in Rush Medical College on last Christmas day. This address will embody the results of many original ex periments, and we trust it will be found interesting to the profes-siongenerally. *Perseverentia omnia vincit, et nil desperandum.*

